# Transcriptional Frameshifting Rescues *Citrobacter rodentium* Type VI Secretion by the Production of Two Length Variants from the Prematurely Interrupted *tssM* Gene

**DOI:** 10.1371/journal.pgen.1004869

**Published:** 2014-12-04

**Authors:** Erwan Gueguen, Norma M. Wills, John F. Atkins, Eric Cascales

**Affiliations:** 1 Laboratoire d'Ingénierie des Systèmes Macromoléculaires (LISM), Institut de Microbiologie de la Méditerranée, CNRS – Aix-Marseille Université, UMR 7255, Marseille, France; 2 Department of Human Genetics, University of Utah, Salt Lake City, Utah, United States of America; 3 Departments of Biochemistry and Microbiology, University College Cork, Cork, Ireland; Max Planck Institute for Terrestrial Microbiology, Germany

## Abstract

The Type VI secretion system (T6SS) mediates toxin delivery into both eukaryotic and prokaryotic cells. It is composed of a cytoplasmic structure resembling the tail of contractile bacteriophages anchored to the cell envelope through a membrane complex composed of the TssL and TssM inner membrane proteins and of the TssJ outer membrane lipoprotein. The C-terminal domain of TssM is required for its interaction with TssJ, and for the function of the T6SS. In *Citrobacter rodentium*, the *tssM1* gene does not encode the C-terminal domain. However, the stop codon is preceded by a run of 11 consecutive adenosines. In this study, we demonstrate that this poly-A tract is a transcriptional slippery site that induces the incorporation of additional adenosines, leading to frameshifting, and hence the production of two TssM1 variants, including a full-length canonical protein. We show that both forms of TssM1, and the ratio between these two forms, are required for the function of the T6SS in *C. rodentium*. Finally, we demonstrate that the *tssM* gene associated with the *Yersinia pseudotuberculosis* T6SS-3 gene cluster is also subjected to transcriptional frameshifting.

## Author Summary

Nonstandard decoding mechanisms lead to the synthesis of different protein variants from a single DNA sequence. These mechanisms are particularly important when the genome length has to be limited such as viral genomes, limited by the available space in the capsid, or to synthesize two different polypeptides that have distinct functional properties. Here, we report that *tssM*, a gene encoded within the *Citrobacter rodentium* Type VI secretion (T6S) gene cluster, is interrupted by a premature stop codon; however, the stop codon is preceded by a slippery site constituted by 11 consecutive adenosines. Reiterative transcription leads to the incorporation of additional nucleotides in the mRNA and therefore restores the original framing. As a consequence, two different TssM variants are created by transcriptional frameshifting, including a full-length 130-kDa protein and an 88-kDa truncated variant. We further show that both forms, and the ratio between these two forms, are required for the function of the transport apparatus. Interestingly, a similar mechanism regulates the synthesis of two TssM variants in *Yersinia pseudotuberculosis.*

## Introduction

The Type VI secretion system (T6SS) is a macromolecular machine widespread in proteobacteria that delivers protein toxins into either eukaryotic or bacterial cells [1]–[5]. The *Vibrio cholerae* T6SS has been shown to inject an effector domain carrying actin cross-linking activity into eukaryotic cells, preventing cytoskeleton rearrangements and allowing the bacteria to escape phagocytosis [6]–[8]. More recently, a number of T6SSs including those of *Pseudomonas aeruginosa*, *V. cholerae*, *Serratia marcescens*, enteroaggregative *Escherichia coli* and *Citrobacter rodentium* have been shown to play antagonistic roles in interbacterial competition including competition occuring during host colonization [5], [9]–[13]. Bacterial preys are killed through the actions of toxins that bear peptidoglycan hydrolase, phospholipase or DNase activities [13]–[15].

For toxin delivery, the T6SS is thought to use a dynamic mechanism resembling that of contractile tailed bacteriophages [3], [4], [16]–[18]. Recent cryo-electron microscopy experiments demonstrated that the T6SS is composed of a cytoplasmic tubular structure anchored to the cell envelope by a membrane complex [18]. The tubular structure is structurally and mechanistically similar to the tail of bacteriophages: the Hcp protein forms hexameric rings that stack on each other to assemble a tube resembling the internal tube of phages and tipped by a trimer of VgrG, which shares a fold similar to the trimeric bacteriophage gp27-gp5 hub – or cell-puncturing – complex [16], [19]–[21]. This internal tube is wrapped into a structure composed of the TssB and TssC subunits [18], [22]. This structure has been shown to be dynamic, as TssB proteins fused to the super-folder Green Fluorescent Protein (sfGFP) form long filaments that cycle between extended and contracted conformations, a mechanism reminiscent of bacteriophage sheaths [18], [23]–[25]. The current model proposes that the mode of action of the T6SS is comparable to that of a crossbow [2]–[4]: the sheath assembles around the Hcp internal tube into an extended conformation. Upon contraction, the internal tube will be propelled towards the target cell allowing the VgrG protein to puncture the host cell and effector delivery. Indeed, recent studies have shown that contraction of the T6SS sheath-like structure coincides with killing of the target bacterial prey [23]–[25].

This “phage-related” complex is anchored to the cell envelope through interactions with membrane components. This membrane complex is composed of the TssL and TssM inner membrane proteins and the TssJ outer membrane lipoprotein [3], [4], [26], [27]. TssM is constituted of three trans-membrane helices with a large C-terminal domain of ∼750 residues protruding into the periplasm [28], [29]. TssM is a central component as it interacts with both TssL and TssJ [28], [29]. The interaction with TssJ has been characterized and involves contacts between a specific loop of the lipoprotein and the 150 last residues of TssM [29]. This interaction is critical for T6SS function as disruption of the TssM-TssJ interaction abolishes Hcp release in the culture supernatant [29]. Although this C-terminal region of TssM is an essential determinant of T6SS function, the TssM protein encoded within the CTS1 T6SS gene cluster of *C. rodentium*, TssM1, is lacking this domain. However, this T6SS is functional as shown by its ability to release the Hcp1 protein in the culture supernatant and to mediate interbacterial killing [30]. Sequence analysis of the *tssM1* gene showed that the stop codon is preceded by a poly-adenosine sequence constituted of eleven consecutive adenosine residues [30], [31]. Poly-A runs have been previously shown to be slippery sites for the RNA polymerase that cause frameshifting by the incorporation of additional adenosine bases into the mRNA during transcription [32]–[35]. Here, using a combination of Western-blot, GFP fluorescence and mass spectrometry analyses, we demonstrate that transcriptional frameshifting occurs at the *tssM1* poly-A run, yielding two TssM1 size variants. We further demonstrate using Hcp secretion and antibacterial competition assays that both forms of TssM1 are required for efficient Type VI secretion in *C. rodentium*. The frequency of frameshifting is ∼20–25% and therefore yields a molecular ratio of 3–4∶1 between the truncated and the full-length variants. This ratio between the two forms is critical as inverting this ratio leads to a non-functional T6SS apparatus. Finally, we show that a similar frameshiting mechanism occurs in the *tssM* gene associated with the *Yersinia pseudotuberculosis* T6SS-3 gene cluster.

## Results

### Sequence analysis of the *C. rodentium* CTS1 *tssM1* gene

**Figure 1:**
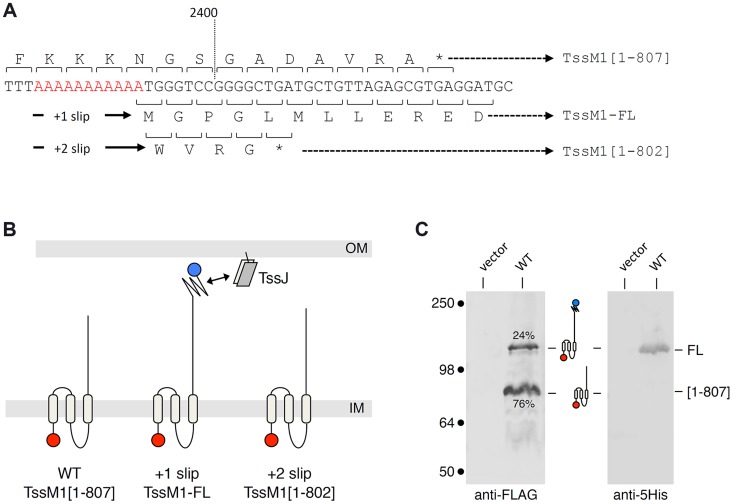
The *C. rodentium tssM1* gene encode two size variants. (A) Representation of the nucleotide sequence of *C. rodentium tssM1*. The 11-nt poly-A tract is shown in red. The codons and the amino-acids resulting from translation of the WT sequence, as well as those produced from +1 or +2 frameshifting are indicated (stops identified by the asterisks) with the size of the corresponding TssM1 variants. (B) Schematic representation of the putative localizations and topologies of the TssM1[1–807] (produced from the WT sequence), TssM1-FL (produced from +1 frameshifting) and TssM1[1–802] (produced from +2 frameshifting) proteins. The locations of the N-terminal FLAG and C-terminal 6×His epitope tags are indicated by red and blue balls respectively (IM, inner membrane; OM, outer membrane). (C) Western-blot analyses of *C. rodentium* cells producing (WT) or not (vector) the TssM1 protein bearing a FLAG-epitope tag at the N-terminus and a 6×His tag upstream the stop codon resulting from +1 frameshifting. Crude extracts from 10^9^ cells were subjected to 10%-acrylamide SDS-PAGE and immunodetection using the anti-FLAG and anti-5His monoclonal antibodies. The positions of the TssM1-FL and -[1–807] variants are indicated on the right, with the schematic representation in the middle. The relative intensities of these two TssM1 variants are indicated (as %). Molecular weight markers (in kDa) are indicated on the left.

Analysis of the *Citrobacter rodentium tssM1* gene sequence (accession numbers: ROD_27701, Gene ID: 8713035) showed that the full-length gene is disrupted by the existence of a premature amber stop codon at position 2,421 (from the start codon) [31]. DNA sequencing of a cloned fragment encompassing this region showed that this amber codon is not a sequencing error. Premature arrest of *tssM1* translation leads to the production of an 807-amino-acid (aa) TssM1 protein (TssM1[1–807]) (Fig. 1A and 1B). Transmembrane helix predictions and sequence alignment of TssM1[1–807] with T6SS TssM proteins of known topology showed that TssM1[1–807] is constituted of the three N-terminal helices but lacks the C-terminal β-domain which has been previously shown to mediate the interaction with TssJ. Interestingly, a sequence corresponding to a β-domain similar to those of canonical TssM proteins is encoded on the +1 reading frame downstream the stop codon of *tssM1[1*–*807]*. Hence, a full-length 1129-aa TssM protein (TssM1-FL) will be produced if +1 frameshifting occurs before the stop codon of *tssM1[1*–*807]* (Fig. 1A and 1B). To test whether frameshifting occurs, we monitored TssM1 production by immunodetection. The 3829-bp sequence of *tssM1-FL* was cloned in an expression plasmid downstream the *tet* promoter. The cloning strategy included the insertion of (i) a FLAG epitope-encoding sequence immediately downstream the start codon and (ii) a 6×His-encoding sequence upstream the stop codon of *tssM1-FL.* Western-blot analyses of cell extracts of *C. rodentium* carrying this expression plasmid demonstrated the production of two proteins of ∼85 and ∼130 kDa, immunostained by the anti-FLAG antibody (Fig. 1C). The molecular weights of these two bands are similar to the expected sizes of TssM1[1–807] (88 kDa) and TssM1-FL (125 kDa). This result suggests that frameshifting occurs in the *tssM1*[1–807] sequence, yielding a full-length TssM1 protein. The production of TssM1-FL was confirmed as the ∼130-kDa protein was detected by anti-5His immunostaining (Fig. 1C). Quantitative analyses showed that the intensity of the high molecular weight protein is ∼1/3 of that of the low molecular weight protein, suggesting that +1 frameshifting occurs with a frequency of ∼25%.

### *C. rodentium tssM1* frameshifting involves a run of adenosines

**Figure 2:**
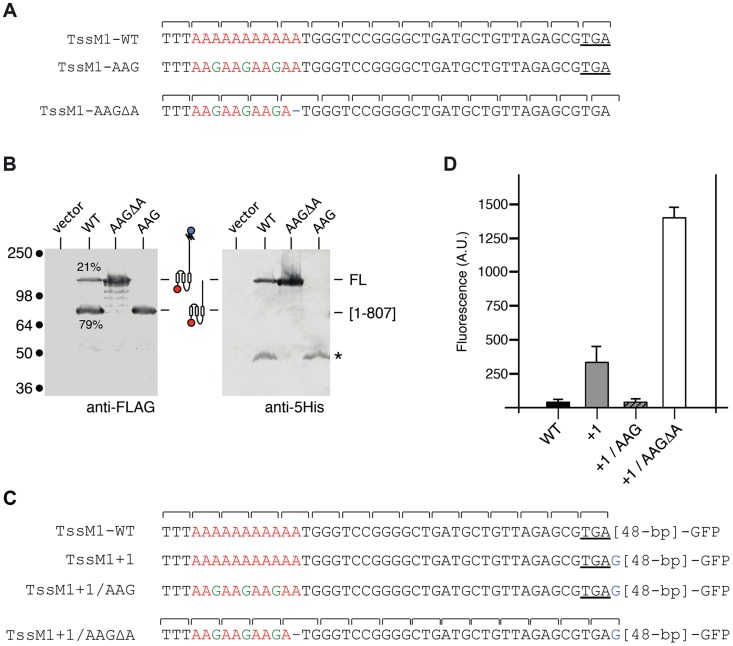
The TssM1 full-length variant is produced through slippage on a poly-A tract. (A) Representation of the nucleotide sequence of *C. rodentium tssM1* and of the mutagenized variants used in panel (B). The poly-A tract is shown in red, the mutagenized nucleotides are shown in green. (B) Western-blot analyses of *C. rodentium* cells producing the indicated TssM1 protein variant bearing a FLAG-epitope tag at the N-terminus and a 6×His tag upstream the stop codon resulting from +1 frameshifting. Crude extracts from 10^9^ cells were subjected to 10%-acrylamide SDS-PAGE and immunodetection using the anti-FLAG and anti-5His monoclonal antibodies. The positions of the TssM1-FL and -[1–807] variants are indicated on the right, with the schematic representation in the middle. The relative intensities of these two TssM1 variants are indicated (as %). Molecular weight markers (in kDa) are indicated on the left. (C) Representation of the nucleotide sequence of *C. rodentium tssM1* constructs used for fluorescence studies shown in panel (D). The poly-A tract is shown in red, the mutagenized nucleotides are shown in green and additional nucleotides are shown in blue. (D) Fluorescence levels (in arbitrary units, A.U.) of cells producing the different TssM1 variants shown in panel (C).

Sequence analysis showed that a stretch of 11 adenosines is localized 28 nucleotides upstream the *tssM1[1*–*807]* stop codon (Fig. 2A). It has been previously showed that poly-adenosine tracts might induce ribosome or RNA polymerase slippage [32]–[35]. To test whether this poly-A tract might be involved in the production of TssM1-FL, we used site-directed mutagenesis to engineer (i) a *tssM1* variant in which the three AAA codons were substituted by three AAG codons (a construct hereof called *tssM1-*AAG) hence disrupting the poly-A tract without modifying the amino-acid sequence and (ii) a *tssM1* variant with a disrupted poly-A and carrying a deletion of the last A to create an artificial +1 frameshift (called *tssM1-*AAGΔA) (Fig. 2A). Western-blot analyses showed that disruption of the poly-A tract prevents frameshifting as only the low molecular weight protein corresponding to TssM1[1–807] was detected by the anti-FLAG antibody. By contrast, only the TssM1-FL variant was detected with both anti-FLAG and anti-5His antibodies when the artificial frameshift construct was analyzed (Fig. 2B). To verify that frameshifting occurs, we used an alternate method by engineering translational fusion of TssM1 to the GFP. The GFP-encoding sequence was inserted 48-pb downstream the premature stop codon of TssM1[1-807] (TssM1-WT, Fig. 2C). This construct serves as negative control for fluorescence (Fig. 2D), as no GFP fusion can be produced (because of the in-frame stop codon). Additional GFP fusions were engineered. In the TssM1+1 construct, an additional nucleotide was inserted between the premature stop codon and the GFP-encoding sequence (Fig. 2C). This construct is a reporter of +1 frameshifting in the natural situation, as the fusion protein will be produced only if +1 frameshifting occurs. Indeed, a significant level of fluorescence compared to the WT sequence can be observed with this construct, demonstrating that frameshifting occurs (Fig. 2D). This frameshifting is dependent on the poly-A run as poly-A disruption by AAA to AAG substitutions in the TssM1+1 construct (TssM1+1/AAG, Fig. 2C) decreases the fluorescence to TssM1-WT levels. Finally, a deletion of the 11th A nucleotide in the TssM1+1/AAG construct yields TssM1+1/AAGΔA (Fig. 2C). In this fusion, no frameshifting can occur but the frame is restored by the additional nucleotide placed after the initial stop codon. Hence, all the produced TssM1 proteins are fused to the GFP and the fluorescence levels reflect the fluorescence if +1 frameshifting occurred with an efficiency of 100%. Comparison of the TssM1+1 and TssM1+1/AAGΔA fluorescence levels showed that the +1 frameshift frequency is ∼20% (Fig. 2D), a value comparable to the frequency calculated from protein immunodetection (Fig. 1C). Taken together, the results from the Western-blot analyses and of the GFP fusions demonstrate that a frameshifting mechanism involving slippage onto a poly-A tract restores the reading-frame of the *tssM1* gene of *C. rodentium* to produce two TssM1 variants: TssM1[1–807] and TssM1-FL.

### *C. rodentium tssM1* reading-frame restoration involves transcriptional slippage

**Figure 3:**
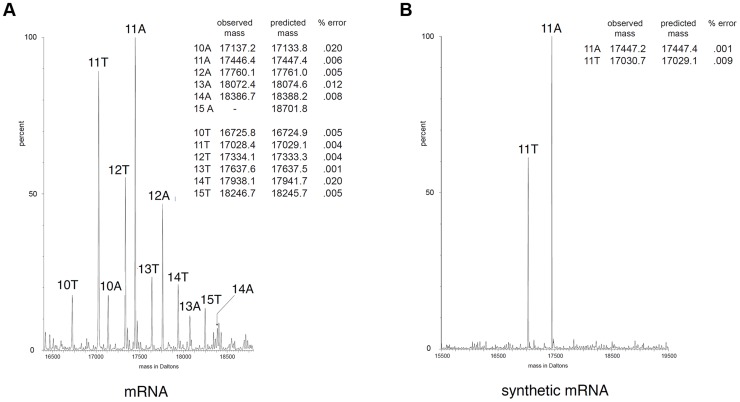
Frameshifting is caused by transcriptional slippage. ESI/MS analyses. Mass spectra of PCR products encompassing the *tssM1* poly-A tract generated by RT-PCR from *C. rodentium* total RNA (mRNA; A) or from a synthetic mRNA (B). The two strands of the PCR products are observable. The molecular mass (in Da) is shown on the *x* axis, and the relative intensity of the signal is indicated on the *y* axis. The number of A or T residues in the homopolymeric run is shown above each peak. The measured and predicted masses (in Da) of each peak are shown in the insert for each panel. The difference between predicted and measured masses is expressed as the percentage error for each peak.

The molecular mechanisms that yield frameshifting have been well described. Translational frameshift could occur during translation of mRNA by ribosomes at specific adenosine repeat, but this mechanism usually requires additional determinants within the mRNA such as Shine-Dalgarno-like sequences or specific mRNA secondary structures close to the stretch of adenosines [33], [35], [36]. However, none of these signals are found at proximity to the *tssM1* frameshifting site. Long stretches of homopolymeric sequences are better known to induce transcriptional slippage, *i.e.*, realignment of the growing RNA to its DNA template within the RNA polymerase. The incorporation of extra, nontemplated, A nucleotide(s) by the RNA polymerase during elongation results in the synthesis of a heterogeneous population of mRNA with different molecular masses [32], [37]. To test whether *tssM1* frameshifting involves ribosome or RNA polymerase slippage, the molecular masses of *tssM1* mRNA products were measured by electrospray ionization mass spectrometry (ESI/MS) as previously reported [37]. Total RNAs were collected from WT *C. rodentium* cells upon activation of the CTS1 T6SS gene cluster using the recombinant strain carrying inducible promoters [30] and *tssM1* cDNA were synthesized and used as template for a PCR reaction. ESI/MS analyses of the PCR products show that products with masses corresponding to molecules bearing 10 to 15 As in the poly-A run were detected (Fig. 3A). The additional adenosines were not incorporated during reverse transcription and PCR amplification as only 11-A PCR products were observed by ESI/MS when a synthetic mRNA corresponding to the region of *tssM1* mRNA subjected to reverse transcription was used as initial template (Fig. 3B). Hence, the presence of the stretch of adenosines induces reiterative transcription by the RNA polymerase and this transcriptional frameshifting restores the *tssM1* reading-frame.

### Both TssM1[1-807] and TssM1-FL are required for CTS1 T6SS function

**Figure 4:**
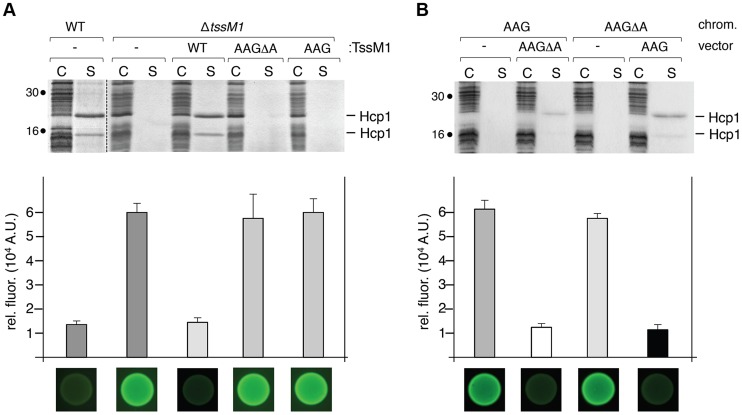
Both TssM1 size variants are required for activity of the CTS1 T6SS. (A) *C. rodentium* WT cells or *ΔtssM1* cells expressing WT *tssM1* (WT; producing both forms of TssM1: TssM1-FL and TssM1[1–807]) or TssM1 variants bearing mutations in the poly-A tract (AAG; producing only the TssM1[1–807] variant) or mutations in the poly-A tract and a deletion of the last nucleotide (AAGΔA; producing only the TssM1-FL variant). (B) *C. rodentium* cells producing the AAGΔA and AAG variants from the chromosome and producing the other variant from the pBAD18 vector. Hcp release assay (top panels). The extracellular proteins were isolated by separating whole cells (C) and the supernatant fraction (S). Proteins were visualized by Coomassie blue staining after 15%-acrylamide SDS-PAGE. Molecular mass markers are indicated on the left. Growth competition assay (bottom panels). Growth competition assay was monitored by mixing fluorescently labeled GFP^+^ *E. coli* W3110 prey cells with the indicated strains of *C. rodentium* as predators. Mixes were spotted onto LB agar supplemented with IPTG (500 µM) and arabinose (2%), and incubated for 14 h at 30°C. The relative fluorescence (upper graph) is expressed in arbitrary units (A.U.) and is the mean of fluorescence levels obtained from three independent experiments (each measured in triplicate). Fluorescent images of the competition assays (obtained with a LI-COR Odyssey imager) are shown below the upper graph.

At a functional level, the most frequent use of frameshifting is to allow the synthesis of a product additional to that of standard decoding. The products can have distinct functions and the ratio between the different products might be important. In other cases frameshifting serves a regulatory function [35]. To address the physiological relevance of the *tssM1* frameshifting for T6SS function, a strain deleted of the *tssM1-FL* gene was constructed. As we did not find conditions in which the CTS1 T6SS gene cluster is expressed, we used in this study recombinant strains in which the expression of the cluster is under the control of inducible promoters, as previously described [30]. CTS1 T6SS function was tested by monitoring Hcp1 release in the culture medium and CTS1-mediated interbacterial killing [30]. As shown previously, the CTS1 was non functional in absence of the *tssM1* gene as shown by the absence of Hcp1 in culture supernatant and by the inability of CTS1 to confer a growth advantage to *C. rodentium* in co-culture with *E. coli* on solid medium (Fig. 4A; [30]). These phenotypes were complemented by the *trans-*expression of *tssM1*, which produces both TssM1[1–807] and TssM1-FL. However, when TssM1[1–807] or TssM1-FL (from *tssM1-AAG* or *tssM1-AAGΔA* respectively) were produced alone, the CTS1 T6SS was not functional: Hcp1 was not released and CTS1-mediated interbacterial killing was abolished (Fig. 4A). These data indicate that both variants of TssM1 are necessary for T6SS function. To further validate these results, we introduced the AAG and AAGΔA substitutions on the chromosome to yield strains producing only one of the TssM1 variants. As shown in Fig. 4B, the CTS1 T6SS was not functional in these two strains; however, when the second variant was expressed *in trans*, Hcp release and CTS1-mediated interbacterial competition were restored to levels comparable to the WT strain. Taken together, these results demonstrate that *C. rodentium* CTS1 T6SS function requires both forms of TssM1, TssM1[1–807] and TssM1-FL, the latter being produced by transcriptional frameshifting.

### Altered ratio between the two TssM1 length variants impair T6SS function

**Figure 5:**
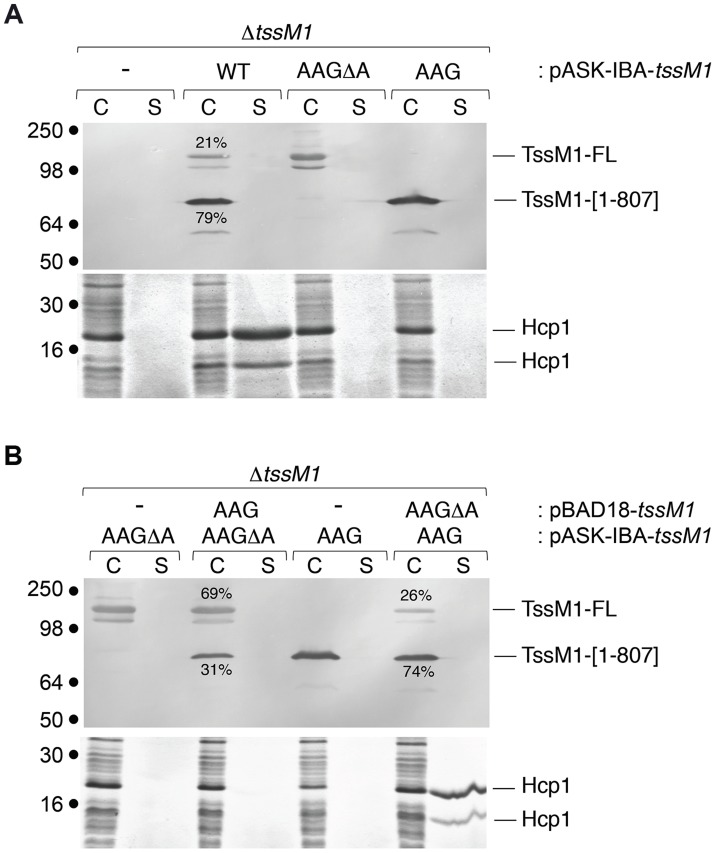
The ratio between the two TssM1 variants is critical for CTS1 T6SS function. Hcp release assays. *C. rodentium* Δ*tssM1* cells expressing WT *tssM1* (WT; producing both forms of TssM1: TssM1-FL and TssM1[1–807]) or TssM1 variants bearing mutations in the poly-A tract (AAG; producing only the TssM1[1–807] variant) or mutations in the poly-A tract and a deletion of the last nucleotide (AAG*Δ*A; producing only the TssM1-FL variant) from the pASK-IBA37+ vector (A) or producing the indicated variants from the pBAD18 and pASK-IBA37+ vectors (B). Extracellular proteins were isolated by separating whole cells (C) and the supernatant fraction (S) from cultures induced with arabinose and anhydrotetracyclin. TssM1 variants were immunodetected using the anti-FLAG monoclonal antibody (upper panels). The relative intensities of the short and long TssM1 variants are indicated (as %). Total proteins were visualized by Coomassie blue staining (lower panels). Molecular mass markers are indicated on the left.

As described above, Western-blot and fluorescence quantifications established that the TssM1[1–807]: TssM1-FL ratio was close to 3–4∶1 (Figs. 1C, 2B and 2C). As both TssM1 variants were required for T6SS function, we asked whether the relative ratio between these two variants is critical. Each form was therefore independently cloned on compatible plasmids: pBAD18 (pBR322 origin) and pASK-IBA37+ (pUC origin). We first verified that producing the natural TssM1 variants in these two vectors (*i.e.*, retaining the natural ratio between the two forms) did not impact the function of the CTS1 T6SS. Production of the two forms from pBAD18 (Fig. 4A) or from pASK-IBA37+ (Fig. 5A) complemented the T6SS-dependent Hcp1 secretion defect of Δ*tssM1* cells. As shown previously for the pBAD18 derivatives, Hcp1 was not released when each length variant was independently produced from pASK-IBA37+ (Fig. 5A). Fig. 5B shows that when TssM1[1–807] was produced at higher levels than TssM1-FL (*i.e.*, the natural situation; Fig. 5B, lanes 7 and 8), Hcp1 was released in the culture supernatant. However, when the ratio was inversed (TssM1-FL produced at higher levels compared to TssM1[1–807]), Hcp1 release was abolished (Fig. 5B, lanes 3 and 4). These data therefore demonstrate that the CTS1 T6SS is functional only when the truncated form of TssM1 is produced in higher amount than the full-length TssM1 variant.

### TssM transcriptional frameshifting is conserved in *Yersinia pseudotuberculosis*

**Figure 6:**
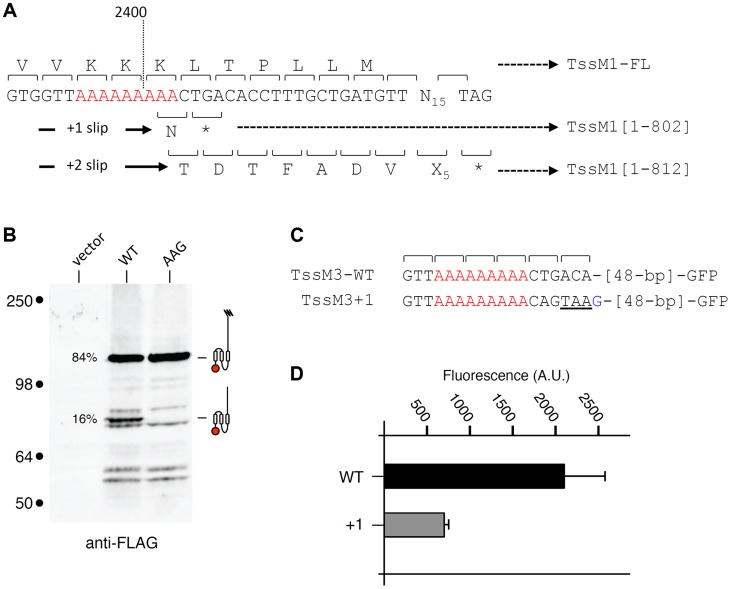
Transcriptional slippage is conserved in *Y. pseudotuberculosis*. (A) Representation of the nucleotide sequence of *Y. pseudotuberculosis tssM3*. The poly-A tract is shown in red. The codons and the amino-acids resulting from translation of the WT sequence, as well as those produced from +1 or +2 frameshifting are indicated with the size of the TssM3 variants. (B) Western-blot analyses of *Y. pseudotuberculosis* cells producing the TssM3 WT protein or a derivative mutated in the poly-A tract (AAG) bearing a FLAG-epitope tag at the N-terminus. Crude extracts from 10^9^ cells were subjected to 10%-acrylamide SDS-PAGE and immunodetection using the anti-FLAG monoclonal antibody. The positions of the TssM3-FL and -[1–802] variants are indicated on the right using the schematic representation. The relative intensities of these two variants are indicated (as %). Molecular weight markers (in kDa) are indicated on the left. (C) Representation of the nucleotide sequence of *Y. pseudotuberculosis tssM3* constructs used for fluorescence studies shown in panel (D). The poly-A tract is shown in red and additional nucleotides are shown in blue. (D) Fluorescence levels (in arbitrary units, A.U.) of cells producing the different TssM1 variants shown in panel (C).

We wondered whether transcriptional frameshifting is a common character among *tssM* genes. T6SS-associated *tssM* gene nucleotide sequences collected from the National Center for Biotechnology and Information (NCBI) were used to identify (i) *tssM* genes with abnormal length and/or (ii) *tssM* genes bearing A or T homopolymeric runs. Interestingly, we found that the *tssM* gene encoded within the *Yersinia pseudotuberculosis* T6SS-3 gene cluster, *tssM3* (accession number: YpsIP31758_1373; gene ID 5385222), has a stretch of 9 As at position 2,394 relative to the start codon (Fig. 6A). The *tssM3* gene encodes a 130-kDa protein. The *tssM3* gene was cloned downstream a FLAG epitope-coding sequence. Western-blot analyses of cell extracts of *Y. pseudotuberculosis* cells producing FLAG-TssM3 revealed a band at ∼80 kDa in addition to the full-length 130-kDa protein (Fig. 6B). This band results from frameshifting as (i) a stop codon is present downstream the poly-A tract in the +1 reading frame (Fig. 6A) and (ii) disruption of the poly-A tract by AAG substitutions of the AAA codons abolished synthesis of the ∼80-kDa protein (Fig. 6B). These data were confirmed by fluorescence levels of GFP fusion proteins: the putative slippery site was active as fusion of the GFP-encoding sequence in the +1 reading frame downstream the poly-A tract (TssM3+1) led to GFP fluorescence (Fig. 6C and 6D). However, although the slippage mechanisms between the *C. rodentium tssM1* and *Y. pseudotuberculosis tssM3* genes are probably similar and allow the synthesis of two variants of different lengths, recoding in *tssM1* leads to the synthesis of the longer variant, whereas recoding in *tssM3* leads to the synthesis of the shorter variant. It is also important to note that the quantification of the anti-FLAG western-blots and the comparison between the fluorescence levels of the TssM3-WT and TssM3+1 GFP fusion constructs showed that the frequency of frameshifting is 15–25%, demonstrating that, in contrast to *C. rodentium* TssM1, the full-length protein is produced at higher levels compared to the truncated form.

## Discussion

In this work, we showed that the sequence of one essential gene of the *C. rodentium* CTS1 T6SS is disrupted by an early stop codon yielding a 88-kDa truncated protein, TssM1[1–807], that lacks a large part of the C-terminal domain required for interaction with other components of the secretion machine; however, we demonstrated that the full-length 125-kDa TssM1 protein is produced during growth. We further demonstrated that transcriptional frameshifting occurs at a slippery site constituted of 11 consecutive adenosine residues, located a few bases upstream the premature stop codon, that induces RNA polymerase infidelity and realignment. This mechanism, although unusual, is not unprecedented. Several examples of RNA editing have been described in viruses, eukaryotes and prokaryotes [33]–[35], [38]–[41]. Frameshifting is particularly frequent in bacteriophages and bacterial insertion sequence (IS) elements [33]–[35]. Well-studied cases are the phage *G* gene which encodes two tail proteins, gpG and gpGT, gpGT arising from translational frameshifting [42], [43] and the *dnaX* gene, which encodes the τ and γ subunits of DNA polymerase III [37], [44]. One striking example is the fusion between *pgk* and *tim*, two different genes that can be fused by transcriptional frameshifting at the 3′ end of *pgk*, yielding a bifunctional chimera protein [38]. The overrepresentation of slippery sites in viruses and bacterial endosymbiots of insects, which have the smallest genomes, suggests that this mechanism helps to condense protein coding in compact genomes [35], [40]. Interestingly, examples of transcriptional frameshifting have been identified in other bacterial secretion systems such as the *Shigella flexneri* Type III secretion system (T3SS), a machinery that mediates entry of the bacterium into epithelial cells. Slippery sites that induce RNA polymerase infidelity have been identified and characterized in the *mxiE* gene that encodes a transcriptional activator of this system as well as in three genes encoding structural components of the T3SS, *mxiA*, *spa13* and *spa33* [45], [46].

The efficiency of the *tssM1* frameshifting was shown to be ∼20-25% leading to a molecular ratio of 3–4∶1 of TssM1[1–807] to TssM1-FL. A similar frequency was measured for *Y. pseudotuberculosis tssM3*. These frequencies are comparable to those measured for transcriptional slippage of the *Shigella flexneri mxiA* (15%), *mxiE* (20–30%), and *spa33* (15%) genes and lower than those measured in the case of *spa13* (55%) [45], [46]. In this study, we have measured the slipppage efficiencies during growth in rich medium (LB). It would be interesting to test whether the slippage frequency is impacted by the growth conditions or by regulatory elements, such as bacteriophage λ N protein, recently shown to influence transcriptional realignment by stabilizing the RNA/DNA hybrid in the RNA polymerase [47].

Using complementation assays, we further showed that both forms of TssM1 are required for T6SS function. Although further experiments are required to better understand what is the specific function of each of these two variants, this situation is reminiscent to that of the phage lambda *G* gene, in which both gpG and gpGT variants are required for efficient assembly of functional tails [48]. In this later case, it was shown that the ratio between gpG and gpGT is also important for formation of phage tails [48]. Similarly, we observed that the ratio between the two TssM1 variants is critical for maintaining a functional CTS1 T6SS. In the natural situation, the shorter variant (TssM1[1–807]) is 3-4 times more abundant than the full-length variant. Inversion of the ratio between the two forms abolishes the function of the CTS1 T6SS.

One additional intriguing result is the observation that a third TssM1 variant of ∼40 kDa, truncated of the N-terminal region, is immunodetected by the C-terminal 6×His epitope (see * in Fig. 2B). This variant therefore corresponds to the C-terminal portion of the TssM1 80-kDa periplasmic domain that is likely retained into the cytoplasm. This variant might be produced from an internal start codon (although sequence analyses did not identify a potential ribosome binding site or an internal start codon) or might result from a proteolytic processing. Experiments are currently carried out to determine how this third variant is produced and to define whether it is necessary for proper assembly or function of the CTS1 T6SS.

Bioinformatic analyses of the T6SS-associated *tssM* genes showed that transcriptional slippery sites are not common as we only identified the *tssM3* gene from *Yersinia pseudotuberculosis* with a poly-A run. Western-blot and fluorescence studies further demonstrated that this site is active as two TssM3 length variants are produced. Slippage occurs with a frequency comparable to that of the *C. rodentium tssM1* situation. Although we have not tested whether these two variants are required for function of the apparatus, it is worthy to note that transcriptional frameshifting in *Y. pseudotuberculosis tssM3* leads to synthesis of a shorter protein whereas *C. rodentium tssM1* slippage leads to synthesis of the full-length protein. As a consequence, and in contrast to *C. rodentium tssM1*, the ratio is in favor of the full-length variant. This is particularly intriguing as our data showed that the ratio between the two variants in *C. rodentium* is critical for the function of the apparatus, and it further suggests that the ratio between the two variants is tailored to fit specific needs during assembly and/or function of the T6SS in different bacteria. In the vast majority of T6SS-associated *tssM* genes, no slippery site can be identified, suggesting that only the full-length protein is produced. However, stable TssM degradation products of ∼85-kDa have been observed by Western-blot analyses of total extracts of WT cells producing TssM in *Agrobacterium tumefaciens* [28] and in enteroaggregative *E. coli*. In these cases, two forms of TssM are therefore produced, the shorter being the result of a degradation mechanism. This observation is particularly fascinating and further experiments are required to understand whether this degradation is a controlled process, whether it is conserved in all TssM proteins, and whether the degradation product is important for the function of the T6SS machines in these bacteria.

## Materials and Methods

### Bacterial strains and growth

Strains used in this study are listed in S1 Table. *Citrobacter rodentium* strains used in this study are derivatives of DBS100 [ATCC51459] (kindly provided by Hervé LeMoual (McGill University, Montreal, Canada)): RLC2 (a spontaneous nalidixic acid resistant variant), RLC55 (a RLC2 derivative in which the promoter as been swapped with a divergent p*lac*-p*ara* promoter) and RLC62 (a Δ*tssM1* derivative of RLC55) [30]. *Yersinia pseudotuberculosis* IP31758 [49] has been kindly provided by Anne Derbise and Elisabeth Carniel (Institut Pasteur, Paris). *Escherichia coli* DH5α, CC118λpir have been used for cloning procedures. *E. coli* W3110 has been used for growth competition assays. MFDpir [50] has been used for mating assays with *C. rodentium*. Strains were routinely grown in Luria-Bertani (LB) broth, or on LB agar plates supplemented with kanamycin (50 µg/mL), chloramphenicol (30 µg/mL) or nalidixic acid (20 µg/mL) when required. MFDpir cells were grown in medium supplemented with 0.3 mM diaminopimelic acid. Induction of the CTS1 T6SS gene cluster expression was performed with IPTG and L-arabinose, as previously described [30].

### Recombinant DNA methods

PCR fragments used for strain and plasmid constructions were amplified with the Phusion high fidelity DNA polymerase (Thermo scientific). Colony PCR amplifications were performed with Taq DNA Polymerase with Standard Taq Buffer (New England BioLabs). Site-directed Quickchange mutagenesis amplifications were done using the Pfu Turbo DNA polymerase (Agilent Technologies). Restriction enzymes were purchased from New England BioLabs. Custom oligonucleotides (listed in S2 Table) were synthesized by Sigma Aldrich.

### Strain construction

The chromosomal poly-A sequence of *tssM1* was modified with the AAG and AAGΔA mutation by allelic exchange using the *sacB*-counter selectable suicide plasmid pSR47S. A *tssM1* fragments of ∼600-bp surrounding the poly-A tract were amplified with oligo pair icmF-crod1-fwd-C/icmF-crod-rev from mutated plasmids (see plasmid construction below) and cloned by TA cloning into pCR2.1 (Invitrogen). After DNA sequencing, BamHI/XbaI fragments from pCR2.1 were inserted into BamHI/SpeI-digested pSR47S, yielding plasmids pRL132 and pRL133. pSR47S derivatives were introduced into *C. rodentium* RLC55 by conjugation using MFDpir as donor and the first recombination event was selected on kanamycin LB plates as previously described [51]. Insertion of pSR47S derivatives was verified by colony PCR. Sucrose counterselection was obtained as described [51] and insertion of the accurate mutation was verified by PCR on purified chromosomal DNA (DNeasy Blood & Tissue kit, Qiagen) and DNA sequencing.

### Plasmid construction

#### *Citrobacter* constructs

Construction of the pBAD-18-kan plasmid carrying the full-length *tssM1* fragment, pRL39, has been previously described [30]. In this construct, the *tssM1* gene is fused to an N-terminal FLAG-coding sequence. Insertion of the 6-His coding sequence before the stop codon of the frameshifted *tssM1* gene was done by cloning a EagI/BglII 448-pb fragment (corresponding to the 3′ end of *tssM1* fused to a 6×His tag sequence, obtained using oligonucleotide icmF-crod1-Cter-6xHis-BglII and icmF-EagI-crod-fwd) into EagI/BglII-linearized pRL39 to yield pRL46 (encoding *flag-tssM1-his_6_* under the control of *areB* promoter). The vector producing the truncated form of TssM1 followed by a 6×His tag (called AAG* was constructed by insertio~ of the 2424-bp of *tssM1* (amplified from pRL39 using SacI-Nter-flag-NdeI-icmF-crod1 a~d icmF-tronq-crod1-Cter-6xHisXbaI) into pBAD18-kan to yield pRL73 (encoding the *tssM1* truncated form fused to an N-terminal FLAG epitope and a C-terminal 6×His sequence).

A series of plasmids derived from pASK-IBA37 (IBA technology) was constructed to follow expression (under the control of the *tet*(promoter) of *tssM1* and its derivatives. The pASK-IBA37+ vector was first modified t introduce the *cat* gene conferring chloramphenicol resistance. The *cat* gene was amplified from pBAD18-Cm using oligonucleotides pIbA-Cm-up and pIBACm-dwn and inserted by restriction-free RF) cloning [52] into the *bla* genm of pASK-IBA37+ to }ield pRL81. *flag-tssM1-his_6_* and flag-tssM1-AAG*-his_6_ fragments were amplified from pRL46 and pRL73 respectively using oligo pairs NheI-flagfwd and XhoI-6xhis-rev and introduced into NheI/XhoI-digested pRL81, yielding pRL102 and pRL109 respectively.

Plasmids carrying *C. rodentium* ‘*tssM1-GFP* fusions were constructed into a pUA66 derivative [53] carrying the *gfpmut2* gene under the control of the constitutive ribosomal *rrnB* promoter (pUA66-*rrnB*). A 90-bp fragment corresponding to *tssM1* encompassing the poly-A run to the premature stop codon (nucleotides 2335-2425 of *tssM1*) was amplified and cloned downstream the ATG stop codon of *gfpmut2* by RF cloning using oligonucleotides pUA66-rrnB-tssM1-5 and pUA66-rrnB-tssM1-3 to yield pRL112 (TssM1-WT). A similar fragment carrying an additional base after the premature stop codon was amplified using pUA66-rrnB-tssM1-5 and pUA66-rrnB-tssM1+1-3 and cloned identically to yield pRL113 (TssM1+1).

Mutations AAG (AAAAAAAAAAA to AAGAAGAAGAA) and AAGΔA (AAAAAAAAAAA to AAGAAGAAGA) were introduced into pBAD-18-Cm, pASK-IBA37+ and pUA66-*rrnB* derivatives by quick-change mutagenesis using oligonucleotide pairs mutLys-icmF-crod1-fwd/mutLys-icmF-crod1-rev and mutLys(-A)-icmF-crod1-fwd/mutLys(-A)-icmF-crod1-rev respectively.

#### *Yersinia* constructs

The *Y. pseudotuberculosis tssM3* gene fused to FLAG and 6×His sequences was amplified using oligonucleotide pair SacI-Nter-FLAG-icmF_1373 and XbaI-Cter-6xHis-icmF_1373 and cloned by TA cloning into pCR2.1. The *flag-tssM3-his6* fragment was then amplified using oligonucleotide pair NheI-flag-fwd and XhoI-6xhis-rev, digested by NheI and XhoI, and cloned into pRL81, to yield pRL103. Mutation of the *tssM3* polyadenosine tract (AAAAAAAAA to AAGAAGAAG) was introduced into pRL103 by quickchange mutagenesis using oligonucleotide pair icmF-1373-mutlys-fwd and icmF-1373-mutlys-rev, yielding pRL106.

Plasmids carrying *Y. pseudotuberculosis* ‘*tssM3-GFP* fusions were constructed into pUA66-*rrnB*. A 63-bp fragment corresponding to *tssM3* encompassing the poly-A run to the premature stop codon (nucleotides 2347–2409 of *tssM3*) was amplified and cloned downstream the ATG stop codon of *gfpmut2* by RF cloning using oligonucleotides pUA66-rrnB-tssM3-5 and pUA66-rrnB-tssM3-3 to yield pRL120 (TssM3-WT). A similar fragment carrying an additional base after the poly-A run was amplified using pUA66-rrnB-tssM3-5 and pUA66-rrnB-tssM3+1-3 and cloned identically to yield pRL122 (TssM3+1).

All the plasmid constructs have been verified by restriction and DNA sequencing.

### Preparation of *C. rodentium* total RNAs, cDNA synthesis and Electrospray Ion Mass Spectrometry (ESI/MS)

Total RNAs were extracted from exponentially-growing *C. rodentium* cells using the RNeasy mini kit (Qiagen). RNA preparations were treated with TURBO DNase (Ambion) to avoid DNA contamination prior to Reverse Transcription (RT)-PCR. The absence of contaminating DNA in the Total RNA preparation was verified by PCR. *tssM1*-specific cDNA encompassing the poly-A run was synthesized from 500 ng of total RNA using oligonucleotide EC955 and the SuperScript II Reverse Transcriptase (Invitrogen). 56-nt PCR products were then amplified from 200 ng of cDNA using primers EC1266 and EC1267 and Phusion DNA polymerase, extracted using the ethanol precipitation procedure and analyzed by electrospray ion mass spectrometry as described previously [37]. As controls for reverse transcription and PCR, PCR products were generated (i) from *C. rodentium* genomic DNA and (ii) from a 56-nt synthetic RNA (GCCGGCUAUUAUGAGGCGUUUAAAAAAAAAAAUGGGUCCGGGGCUGAUGCUGUUAG) (Eurogentec).

### Hcp release and antibacterial competition assay

The Hcp1 release assay has been performed as previously described [30]. The antibacterial growth competition assay has been performed as previously described, using the *E. coli* K-12 strain W3110 bearing the pUA66-rrnB plasmid (Kan^R^ and strong and constitutive GFP fluorescence) as prey [30]. Briefly, *Citrobacter* and *E. coli* cells were mixed to a 4∶1 ratio and the mixture was spotted onto prewarmed dry plates and incubated for 16 hours at 30°C. Fluorescent images were taken using a LI-COR Odyssey imager and the relative fluorescence was measured after resuspension of the bacterial cells using a TECAN Infinite M200 microplate reader.

### Fluorescence assay

*C. rodentium cells* carrying the pUA66-*rrnB* plasmid derivatives were grown in LB at 37°C to an OD_600_ of ∼1 and normalized to an OD_600_ of 0.5. Triplicates of 150 µl were transferred into wells of a black 96-well plate (Greiner) and the absorbance at 600 nm and fluorescence (excitation: 485 nm; emission: 530 nm) were measured with a TECAN infinite M200 microplate reader. The experiments were done in triplicate and the relative fluorescence was expressed as the intensity of fluorescence divided by the absorbance at 600 nm, after subtracting the values of a blank sample.

### Miscellaneous

Gene expression from pBAD18 and pASK-IBA37(+) derivatives was induced in exponentially growing cultures (OD_600_∼0.5) using arabinose (0.2%) for 1 hour and AHT (5 or 10 ng/ml) for 30 minutes respectively. For Western-blot analyses, cells were resuspended in Laemmli buffer (2×10^11^ cells/ml). Proteins were separated by SDS-PAGE analyses and transferred onto nitrocellulose membranes. Immunoblots were probed with anti-5His (Qiagen) or anti-FLAG (Sigma) antibodies, and anti-mouse secondary antibodies coupled to fluorophores. Immunodetection and band density analyses were performed using a LI-COR Odyssey imager.

## Supporting Information

Table S1 Strains and plasmids used in this study.
(DOCX)

Table S2 Oligonucleotides used in this study.
(DOCX)
