# Supplementary material for: Transcriptional Frameshifting Rescues Citrobacter rodentium Type VI Secretion by the Production of Two Length Variants from the Prematurely Interrupted tssM Gene
Source: PLoS Genet. 2014 Dec 4;10(12):e1004869. doi: 10.1371/journal.pgen.1004869 (PMC4256274; doi:10.1371/journal.pgen.1004869)
Supplement: Table S1 — Strains and plasmids used in this study. (DOCX) [file pgen.1004869.s001.docx]

Transcriptional frameshifting rescues *Citrobacter rodentium* Type VI secretion by the production of two length variants from the prematurely interrupted *tssM* gene.

E. Gueguen, N.M. Wills, J.F. Atkins, E. Cascales

**Supplemental Table S1. Strains and plasmids used in this study.**

**STRAINS**

**Name Genotype or feature(s) Reference or source**

*Citrobacter rodentium* DBS100 ATCC 51459 derivatives

RLC2 Nal^R^ derivative of *C. rodentium* DBS100 (ATCC 51459) [1]

RLC55 RLC2 *P_cts1V_::P_tac_*, *P_cts1C_::araC-P_BAD_* [1]

RLC62 RLC55 Δ*tssM1* [1]

RLC90 RLC55 *tssM1(AAG*Δ*A)* This study

RLC91 RLC55 *tssM1(AAG)* This study

*Yersinia pseudotuberculosis IP31758 derivatives*

IP31758 Wild-type *Yersinia pseudotuberculosis* [2]

RL31758-4 Nal^R^ derivative of IP31758 [2]

*Escherichia coli* strains

DH5α *fhuA2* Δ*(argF-lacZ)U169 phoA glnV44* Φ*80* Δ*(lacZ)M15* *gyrA96 recA1 relA1 endA1 thi-1 hsdR17* Laboratory collection

W3110 F^-^ lambda^-^ IN(*rrnD-rrnE*)1 *rph*-1 Laboratory collection

CC118λpir *Δ(ara-leu) araD ΔlacX74 galE galK phoA20 thi-1 rpsE rpoB argE* (Am) *recA1* λpir lysogen Laboratory collection

MFD λpir MG1655 RP4-2-Tc::[ΔMu1::*aac(3)IV*-Δ*aphA*-Δ*nic*35 ΔMu2::*zeo*] Δ*dapA*::(*erm*-*pir*) Δ*recA*  [3]

**PLASMIDS**

***C. rodentium* plasmids.**

pCR2.1 TA cloning vector Invitrogen

pSR47S KmR R6K *ori*, *mob*+ (RP4) *sacB*+ Merriam *et al.*, 1997

pRL132 500-bp *tssM1(AAG*Δ*A)* fragment in pSR47S This study

pRL133 500-bp *tssM1(AAG)* fragment in pSR47S This study

pBAD18-Kan KmR ColE1 *ori*, *araBp* expression vector [5]

pRL39 *araBp-flag-tssM1* in pBAD18-kan [1]

pRL46 *araBp-flag-tssM1-his_6_* in pBAD18-kan This study

pRL73 *araBp-flag-tssM1(AAG*)-his_6_* in pBAD18-kan This study

pRL71 *araBp-flag-tssM1(AAG)-his_6_* in pBAD18-kan This study

pRL72 *araBp-flag-tssM1(AAG*Δ*A)-his_6_* in pBAD18-kan This study

pASK-IBA37+ Amp^R^, f1 ori, *tetp* expression vector IBA Technologies

pBAD18-Cm CmR ColE1 *ori*, *araBp* expression vector [5]

pRL81 pASK-IBA37+ with *cat* (Cm^R^) gene inserted into the *bla* gene This study

pRL102 *C. rodentium* *flag-tssM1-his_6_* downstream P_tet_ in pRL81 This study

pRL104 *C. rodentium* *flag-tssM1(AAG*Δ*A)-his_6_* downstream P_tet_ in pRL81 This study

pRL105 *C. rodentium* *flag-tssM1(AAG)-his_6_* downstream P_tet_ in pRL81 This study

pRL109 *C. rodentium* *flag*-*tssM1(AAG*)-his_6_* downstream P_tet_ in pRL81 This study

pUA66 KmR sc101 *ori*, promoterless version of the GFP reporter plasmid [6]

pUA66-rrnb *rrnBp::gfpmut2* transcriptional fusion in pUA66 [6]

pRL112 pUA66-rrnB, fusion *‘tssM1’-gfpmut2* (TssM1) This study

pRL113 pUA66-rrnB, fusion *‘tssM1’-gfpmut2* with additional nucleotide (TssM1+1) This study

pRL114 pRL113 with AAGAAGAAGA (instead of AAAAAAAAAAA) substitution This study

pRL116 pRL113 with AAGAAGAAGAA (instead of AAAAAAAAAAA) substitution This study

***Y. pseudotuberculosis* plasmids.**

pRL84 *flag-tssM3-his_6_* from *Y. pseudotuberculosis* IP31758 in pCR2.1 This study

pRL103 *flag-tssM1-his_6_* from *Y. pseudotuberculosis* IP31758 downstream P_tet_ in pRL81 This study

pRL106 pRL103 with AAGAAGAAG (instead of AAAAAAAAA) substitution This study

pRL118 pUA66-rrnB, fusion *‘tssM3’-gfpmut2* (TssM3+1) This study

pRL120 pUA66-rrnB, fusion *‘tssM3’-gfpmut2* (TssM3-WT) This study

**REFERENCES**

[1] Gueguen E, Cascales E (2013) Promoter swapping unveils the role of the *Citrobacter rodentium* CTS1 Type VI secretion system in interbacterial competition. Appl Environ Microbiol 79: 32-8.

[2] Gueguen E, Durand E, Zhang XY, d’Amalric Q, Journet L, et al. **(**2013) Expression of a *Yersinia pseudotuberculosis* Type VI secretion system is responsive to envelope stresses through the OmpR transcriptional activator. PLoS One 8: e66615*.*

[3] Ferrières L, Hémery G, Nham T, Guérout AM, Mazel D, et al. (2010) Silent mischief: bacteriophage Mu insertions contaminate products of *Escherichia coli* random mutagenesis performed using suicidal transposon delivery plasmids mobilized by broad-host-range RP4 conjugative machinery. J Bacteriol 192: 6418-27.

[4] Merriam JJ, Mathur R, Maxfield-Boumil R, Isberg RR (1997) Analysis of the *Legionella pneumophila fliI* gene: intracellular growth of a defined mutant defective for flagellum biosynthesis. Infect Immun. 65: 2497-501.

[5] Guzman LM, Belin D, Carson MJ, Beckwith J (1995) Tight regulation, modulation, and high-level expression by vectors containing the arabinose PBAD promoter. J Bacteriol 177: 4121-30.

[6] Zaslaver A, Bren A, Ronen M, Itzkovitz S, Kikoin I, et al. (2006) A comprehensive library of fluorescent transcriptional reporters for *Escherichia coli*. Nat Methods. 3**:** 623-8.
