# Supplementary material for: Transcriptional Frameshifting Rescues Citrobacter rodentium Type VI Secretion by the Production of Two Length Variants from the Prematurely Interrupted tssM Gene
Source: PLoS Genet. 2014 Dec 4;10(12):e1004869. doi: 10.1371/journal.pgen.1004869 (PMC4256274; doi:10.1371/journal.pgen.1004869)
Supplement: Table S2 — Oligonucleotides used in this study. (DOCX) [file pgen.1004869.s002.docx]

Transcriptional frameshifting rescues *Citrobacter rodentium* Type VI secretion by the production of two length variants from the prematurely interrupted *tssM* gene.

E. Gueguen, N.M. Wills, J.F. Atkins, E. Cascales

**Supplemental Table S2. Oligonucleotides used in this study.**

**Oligonucleotides for strain and plasmid construction. ^a,b,c^**

**Primer Sequence (5’-3’) Strain or plasmid**

Construction of *C. rodentium tssM1* poly-A run mutant strains.

icmF-crod1-fwd-C ATCTGACCCGGCTTTTTGC RLC90 (pRL132), RLC91 (pRL133)

icmF-crod-rev GATTCACCAGCGGCGAATG RLC90 (pRL132), RLC91 (pRL133)

Construction of the pASK-IBA37+ chloramphenicol resistant vector derivative.

pIBA-CM-up **GCCTGTAGCAATGGCAACAACGTTGC**TTATTATCACTTATTCAG GCGTAGCACC pRL81

pIBA-CM-dwn **GAGTAAGTAGTTCGCCAGTTAATAGTTTGC**AGAGGTTCCAACTTT CACCATAATG pRL81

Cloning of *C. rodentium tssM1* in expression vectors.

icmF-crod1-Cter-6xHis-BglII **CTAGAGATCTCCTCGGATTTTA**GTGATGGTGATGGTGATGATAT TCGCCAGGGCAGCGGAAATCT pRL46

icmF-EagI-crod-fwd GGAGAGAACGGCCGAAAATTAGC pRL46

SacI-Nter-flag-NdeI-icmF-crod1 AAGGAGCTCAACGAGGACGACAATGGACTACAAAGATGACGACGATAA ACATATGTCTTATTTAAATAGAGTCTTCACCACGCGCTTTATG pRL73

icmF-tronq-crod1-Cter-6xHis-XbaI TCCTCTAGATCAGTGATGGTGATGGTGATGCGCTCTAACAGC ATCAGCCCCGG pRL73

NheI-flag-fwd ATGGCTAGCGACTACAAAGATGACGACGATAAA pRL102, pRL103, pRL109

XhoI-6xHis-rev ATACTCGAGTCAGTGATGGTGATGGTGATG pRL102, pRL103, pRL109

Construction of the *C. rodentium tssM1* pUA66-rrnB derivatives

pUA66-rrnB-tssM1-5 **GGATCCTCTAGATTTAAGAAGGAGATATACATATG**GGCGCCA TTCCCGGCCTTTTTACC pRL112, pRL113

pUA66-rrnB-tssM1-3 **CTCCAGTGAAAAGTTCTTCTCCTTTACT**TCACGCTCTAACAGCA TCAGCCCCG pRL112

pUA66-rrnB-tssM1+1-3 **CTCCAGTGAAAAGTTCTTCTCCTTTACT***C*TCACGCTCTAACAGCA TCAGCCCCG pRL113

Site-directed mutagenesis of plasmid-borne *C. rodentium tssM1*.

mutLys-icmF-crod1-fwd GAGGCGTTTAA***G***AA***G***AA***G***AATGGGTCCGGGGCTGATGCTGTTA pRL71, pRL105, pRL114

mutLys-icmF-crod1-rev GACCCATT***C***TT***C***TT***C***TTAAACGCCTCATAATAGCCGGCG pRL71, pRL105, pRL114

mutLys(-A)-icmF-crod1-fwd GAGGCGTTTAA***G***AA***G***AA***G***ATGGGTCCGGGGCTGATGCTGTTA pRL72, pRL104, pRL116

mutLys(-A)-icmF-crod1-rev GACCCATT***C***TT***C***TT***C***TAAACGCCTCATAATAGCCGGCG pRL72, pRL104, pRL116

Cloning of *Y. pseudotuberculosis tssM3* in expression vectors.

SacI-Nter-FLAG-icmF_1373 CGGGAGCTCAGGAGGAATTCACCATGGACTACAAAGATGACG ACGATAAATCTTATTTGAATCGATTGTTTTCTGGCTG pRL84

XbaI-Cter-6xHis-icmF_1373 TCCTCTAGATTAGTGATGGTGATGGTGATGCCAAATACCATTC ATGTCATTGACACTG pRL84

Site-directed mutagenesis of plasmid-borne *Y. pseudotuberculosis tssM3*.

icmF-1373-mutlys-fwd CCATGAGGTGGTTAA***G***AA***G***AA***G***CTGACACCTTTGCTGATGTTACTTCAAG pRL106

icmF-1373-mutlys-rev CAAAGGTGTCAG***C***TT***C***TT***C***TTAACCACCTCATGGTAACCCGCGTAGGTATAAAG pRL106

Construction of the *Y. pseudotuberculosis tssM3* pUA66-rrnB derivatives

pUA66-rrnB-tssM3-5 **GGATCCTCTAGATTTAAGAAGGAGATATACATATG**GAAGGGATC CCAGGGCTTTATACCTACG pRL118, pRL120

pUA66-rrnB-tssM3+1-3 **CTCCAGTGAAAAGTTCTTCTCCTTTACT**GTTTTTTTTTAACCACCT CATGGTAACCCGC pRL118

pUA66-rrnB-tssM3-3 **CTCCAGTGAAAAGTTCTTCTCCTTTACT**TGTCAGTTTTTTTTTAAC CACCTCATGGTAACC pRL120

**Oligonucleotides for DNA sequencing.**

DNA sequencing of the *C. rodentium* *tssM1* region.

icmF-crod1-fwd-A ACGACGCTATCTGGCTTGAC

icmF-crod1-fwd-B TAAGTCGCCGGAGGTGATG

icmF-crod1-fwd-D GCTTATGCCAGCATGGAAAAG

Verification of the *tssM1* sequence in pRL81

IBAProm GAGTTATTTTACCACTCCCTATC

IBA-Rev CGCAGTAGCGGTAAACGGCAG

DNA sequencing of the *Y. pseudotuberculosis* *tssM3* region.

icmF_1373-B TGCTTGAGGATCGTATCAGCAATAATATG

icmF_1373-D GTTGAAGCTTTATTTGATGCTGACGG

icmF_1373-E CCCCGGGGGGCAATAATCTTTC

**Oligonucleotides for Reverse-Transcription and PCR amplification of *C. rodentium tssM1* mRNA.**

EC955 CGCTAATTTTCGGCCGTTCTCTCC

EC1321 CACAGGAAGATCGGTCCATT

EC1322 AATTTATTGGCGGTTTGCAG

EC1266 GCCGGCTATTATGAGGCG

EC1267 CTAACAGCATCAGCCCCG

^a^ Tag-encoding sequences and restriction sites underlined

^b^ Sequence complementary to target vector in bold letters.

^c^ Mutagenized bases in bold italics.
